# Association between systemic immune inflammation index and cataract incidence from 2005 to 2008

**DOI:** 10.1038/s41598-024-84204-7

**Published:** 2025-01-02

**Authors:** Xiang Li, Guo-lei Du, Shi-Nan Wu, Yi-qing Sun, Si-Qi Zhang, Zhi-Jie Zhang, Jia-feng Tang

**Affiliations:** 1 Eye Institute & Affiliated Xiamen Eye Center, School of Medicine, Xiamen University, XiaMen, China; 2 Chongqing Key Laboratory of Development and Utilization of Genuine Medicinal Materials in Three Gorges Reservoir Area, Chongqing Three Gorges Medical College, No. 366, Tian Xing Rd, Bai’anba, Wanzhou, chongqing China; 3 Weihai Institute for Bionics-Jilin University, Weihai, China; 4 The First Affiliated Hospital of Xi’an Jiao Tong University, Shaanxi, 710061 China; 5 Kunming Medical University, Kunming, 650500 Yunnan China

**Keywords:** Systemic immune-inflammation index, Cataracts, NHANES, Systemic inflammation, Risk assessment, Eye diseases, Public health, Biomarkers, Diseases, Risk factors

## Abstract

The objective of this study is to investigate the association between the Systemic Immune-Inflammation Index (SII) and cataracts. This cross-sectional study analyzed data from the 2005–2008 NHANES to examine the relationship between the SII and cataract prevalence. Covariates included age, race/ethnicity, gender, education level, marital status, Body Mass Index (BMI), smoking, alcohol consumption, hypertension, hyperlipidemia, and diabetes. Multivariable logistic regression was used to assess the association, while spline curve fitting explored potential non-linear relationships. Threshold analysis identified critical inflection points. To address age-related bias, Propensity Score Matching (PSM) was performed, aligning cataract patients with comparable non-cataract individuals for further evaluation. Our study included 3,623 participants, of whom 730 (20.15%) were diagnosed with cataracts. After adjusting for all covariates, multivariable logistic regression analysis demonstrated that elevated levels of the SII were significantly associated with increased odds of cataracts (Model1: OR = 1.56; 95%CI [1.33–1.85]; Model2: OR = 1.55; 95%CI [1.32–1.84]; Model3: OR = 1.57; 95%CI [1.33–1.86]). In the spline curve fitting model, the relationship between ln-SII and cataract prevalence was non-linear (P < 0.001), with a critical inflection point identified at an SII of 428.38. SII levels remained significantly associated with cataract prevalence following PSM adjustments (Model 1: OR = 1.48; 95% CI [1.21–1.80]; Model 2: OR = 1.48; 95% CI [1.21–1.80]; Model 3: OR = 1.46; 95% CI [1.20–1.78]). Elevated SII levels are associated with a higher prevalence of cataracts, underscoring the pivotal role of systemic inflammation in cataract development. These findings indicate that SII could serve as a valuable biomarker for assessing cataract risk, further emphasizing the significance of managing systemic inflammation as a potential strategy for cataract prevention.

## Introduction

The lens, positioned behind the iris yet in front of the vitreous body and retina, plays a crucial role in directing light onto the retina. Originating from ectodermal tissue, it comprises epithelial cells that produce lens fibers, causing the lens to thicken over time. Cataracts, characterized by reduced lens clarity, impair vision, decrease contrast sensitivity, alter color perception, and cause glare^1–3^. The World Health Organization reports that cataracts represent approximately 46% of the nearly 180 million global cases of visual impairments^3^. Although treatable, cataracts remain a leading cause of vision loss worldwide, posing significant public health challenges^4,5^. Factors such as aging, smoking, diabetes, and exposure to ultraviolet light contribute to the development of age-related cataracts^6^. Although cataract surgery can significantly improve vision, its affordability and the limited availability of surgeons in some regions continue to pose challenges^7^.

Immune cells play a crucial role in the systemic inflammatory response, which is implicated in a variety of diseases. Researchers have found that the combined count of lymphocytes, neutrophils, and platelets in peripheral blood provides a more accurate indication of inflammatory status^8,9^. Recent ophthalmologic studies have demonstrated a significant association between Systemic Immune-Inflammation Index (SII) and primary open-angle glaucoma^10^. Additionally, inflammation and immune responses are increasingly recognized as key factors in cataract development. Chronic inflammation induces oxidative stress, which promotes lens protein modification and ultimately leads to lens opacity^11^. Moreover, immune responses involving cytokines and inflammatory mediators have been implicated in the progression of cataracts.

Initially, SII was identified as a prognostic marker for gastrointestinal tumors in elderly patients^12^. Subsequent studies have also linked it to the occurrence of pseudophakic cystoid macular edema (PCME) following uncomplicated phacoemulsification cataract surgery in patients without risk factors, highlighting its potential as a predictive biomarker for PCME. This suggests its utility in enhancing clinical assessments and refining risk stratification^13^. However, the relationship between SII and cataracts remains unexplored. To address this gap, our research team conducted a population-based cross-sectional analysis using NHANES data to investigate the potential association between SII and cataract prevalence in adults.

## Materials and methods

### Data source and subject selection

**Fig. 1:**
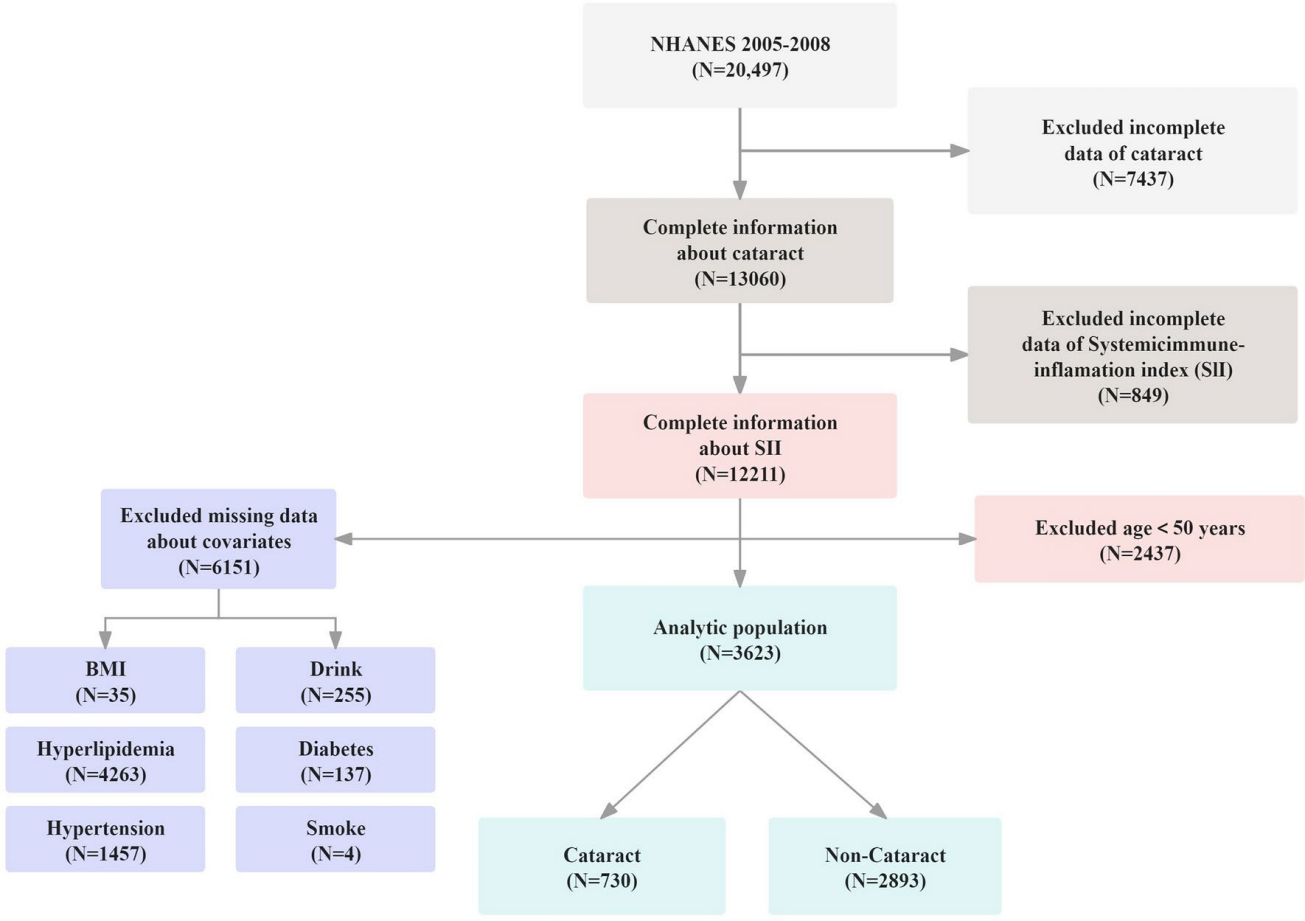
Screening process of the included studies.

The NHANES is a cross-sectional study that collects comprehensive data on the health and nutritional status of U.S. households^14^. It employs a complex, stratified, multistage probability cluster sampling method to ensure an accurate representation of the U.S. population. For further insights into NHANES’s detailed methodology, please refer to their website at http://www.cdc.gov/nchs/nhanes/index.htm. Ethical clearance for the study was granted by the National Center for Health Statistics’ Ethics Committee, with all subjects providing written informed consent^8^. Our study excluded individuals with missing SII data, cataract information, or other necessary covariate details. Given the strong correlation between cataracts and age, we further excluded participants under the age of 50 to enhance the reliability and validity of our findings. This approach targeted an age group with a higher prevalence of cataracts, thereby minimizing the confounding effect of age on the association of the SII with cataract, and the effect of age on all potential confounders included in the model. Ultimately, the study population comprised 3,623 individuals, with the sample selection process illustrated in Fig. 1.

### Cataract identification criteria

According to the NHANES Vision Procedures Manual^15^, participants aged 20 and older were asked whether they had undergone cataract surgery. To minimize confounding factors associated with age, participants under the age of 50 were further excluded. Given the high accessibility and low barriers to cataract surgery in the United States, self-reported cataract surgery is considered a surrogate indicator for clinically significant cataracts. Participants completed a questionnaire (VIQ071: 2005–2008), which included the question, "Have you ever had cataract surgery?" An affirmative response was taken as indicative of cataracts^16^. This method of identifying cataracts is consistent with the methodologies used in previous studies^16,17^.

### The definition of systemic immune-inflammation index

The Complete Blood Count (CBC) metrics employ the Beckman Coulter methodology, which integrates counting and sizing techniques, an automated system for diluting and mixing samples, and a single-beam photometer for measuring hemoglobin levels. White Blood Cell (WBC) differentials utilize VCS technology. Positioned within the NHANES mobile examination center (MEC), the Beckman Coulter DxH 800 instrument conducts CBC analyses on participant blood samples, providing a comprehensive cell distribution profile. The SII is derived from these three circulating immune cell types and is calculated using the formula: platelets (PC) × neutrophils (NC) / lymphocytes (LC) ^18^.

### Covariates assessment

Demographic variables such as age, race/ethnicity, gender, education level, marital status, and Body Mass Index (BMI) were selected as covariates in our study. This demographic information was collected via computer-assisted personal interviews^19^. The comorbidities considered included hypertension, hyperlipidemia, and diabetes mellitus. Hypertension was defined as either a doctor’s diagnosis, the use of antihypertensive medication, or having a systolic blood pressure ≥ 140 mmHg or diastolic blood pressure ≥ 90 mmHg. Hyperlipidemia was identified in participants with a doctor’s diagnosis, those taking lipid-lowering medications, or those with a total cholesterol level ≥ 240 mg/dL during NHANES assessments. Diabetes mellitus was determined by a doctor’s diagnosis, the use of glucose-lowering medication or insulin, or an HbA1c level ≥ 6.5% during NHANES testing. In terms of lifestyle, the covariates included were smoking and alcohol consumption. Smoking was categorized into two groups: non-smokers (those who have never smoked or have smoked fewer than 100 cigarettes in their lifetime) and smokers (those who have smoked at least 100 cigarettes in their lifetime)^20^. Similarly, alcohol consumption was divided into two categories: non-drinkers (those who have never consumed alcohol or have consumed fewer than 12 drinks in their lifetime) and drinkers (those who have consumed at least 12 drinks in their lifetime)^21^.

### Statistical methods

Data analysis was performed using R2 and EmpowerStats software, developed by X&Y Solutions, Inc., based in Boston, MA, and available at http://www.empowerstats.com. This analysis accounted for the complex sampling framework of NHANES by incorporating sampling weights, strata, and primary sampling units. Continuous variables were presented as means ± standard errors (SE), and categorical variables as percentages ± SE. Chi-square tests or T-tests were utilized to assess demographic differences.

The SII data exhibited a right-skewed distribution, necessitating a natural logarithm transformation for statistical analysis. Logistic regression models were utilized to assess the association between SII levels and cataract risk. Model 1 adjusted for age, gender, race, and BMI. Model 2 included additional adjustments for education level, marital status, smoking, and alcohol consumption. Model 3, building on Model 1, also accounted for diabetes, hypertension, and hyperlipidemia. Quantile regression analyses were conducted to further explore these associations. The logistic regression results were visually represented through forest plots, and smooth curve fitting was utilized to investigate potential relationships between SII levels and cataract risk. Analysis of threshold and saturation effects were performed to identify the optimal inflection point. In order to mitigate potential age-related effects on the outcomes, cataract patients and non-cataract individuals were matched in a 1:1 ratio based on average age in the propensity score matching (PSM) analysis. Statistical significance was established at p-values less than 0.05.

## Results

### Description of baseline information of the study sample

The study encompassed a total of 3,623 participants, including 2,893 individuals without cataracts and 730 who were diagnosed with cataracts following screening. The characteristics of these participants are summarized in Table 1.

**Table 1:** Characteristics of participants stratified by cataract from NHANES 2005–2008.

Variables	Non-Cataract	Cataract	p-Value
N	2893	730	
Age (years)	63.79 ± 9.02	74.28 ± 8.19	< 0.001
BMI (kg/m^2^)	29.46 ± 6.29	28.43 ± 6.05	< 0.001
SII(× 10^3^/mm^3^)	563.23 ± 333.37	703.04 ± 474.09	< 0.001
Gender, n(%)			0.021
Male	1454 (50.26%)	332 (45.48%)	
Female	1439 (49.74%)	398 (54.52%)	
Race			< 0.001
Mexican	400 (13.83%)	56 (7.67%)	
Other Hispanic	208 (7.19%)	30 (4.11%)	
Non-Hispanic white	1601 (55.34%)	541 (74.11%)	
Non-Hispanic black	603 (20.84%)	84 (11.51%)	
Other race	81 (2.80%)	19 (2.60%)	
Education, n(%)			< 0.001
Less than 9th grade	373 (12.89%)	129 (17.67%)	
9–11Th grade (includes 12th grade with no diploma)	403 (13.93%)	104 (14.25%)	
High school grad/ged or equivalent	733 (25.34%)	196 (26.85%)	
Some college or AA degre	726 (25.10%)	176 (24.11%)	
College graduate or above	658 (22.74%)	125 (17.12%)	
Marital Status, n(%)			< 0.001
Married or living with partner	1872 (64.71%)	392 (53.70%)	
Unmarried or other	1021 (35.29%)	338 (46.30%)	
Drink, n(%)			< 0.001
Yes	1943 (67.16%)	437 (59.86%)	
No	950 (32.84%)	293 (40.14%)	
Hypertension, n(%)			< 0.001
Yes	1562 (53.99%)	455 (62.33%)	
No	1331 (46.01%)	275 (37.67%)	
Hyperlipemia, n(%)			< 0.001
Yes	1552 (53.65%)	515 (70.55%)	
No	1341 (46.35%)	215 (29.45%)	
Diabetes, n(%)			< 0.001
Yes	532 (18.39%)	534 (73.15%)	
No	2361 (81.61%)	196 (26.85%)	
Smoke, n(%)			< 0.001
Yes	1718 (59.38%)	494 (67.67%)	
No	1175 (40.62%)	236 (32.33%)	

Mean ± SD for continuous variables: the p-value was calculated by a weighted linear regression model. % for categorical variables: the p-value was calculated by a weighted chi-square test. BMI, body mass index.

The average age of participants was 65.9 years, consisting of 1,786 males (49.30%) and 1,837 females (50.70%). Participants who had undergone cataract surgery were predominantly older, unmarried females with lower levels of education. Additionally, it was observed that patients with a history of smoking or alcohol consumption were more susceptible to developing cataracts. Furthermore, participants diagnosed with hypertension, hyperlipidemia, or diabetes exhibited a higher incidence of cataract formation. Table 1 demonstrates that individuals with cataracts presented higher SII scores, corroborating our initial hypothesis.

### Association between SII and cataract

**Fig. 2:**
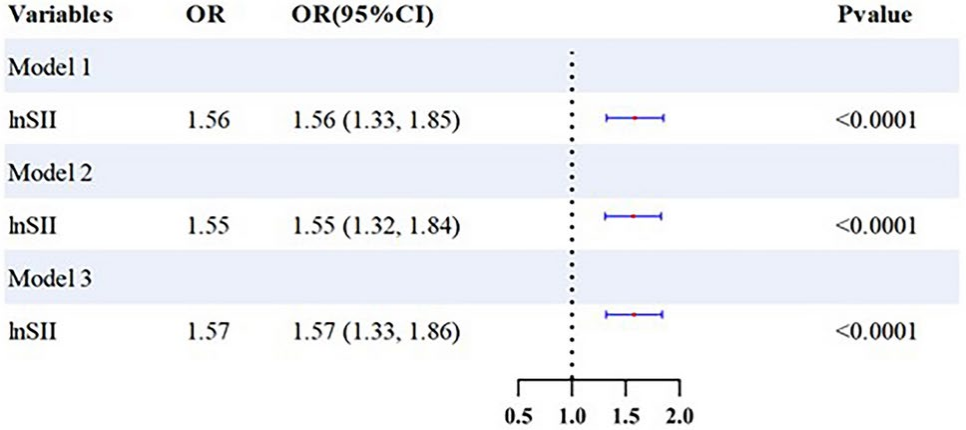
Forest plot of logistic regression results. *Note*: Model 1: adjusted for Gender; Age; Race; BMI. Model 2: adjusted for Gender; Age; Race; BMI; Education; Marital Status; Smoke; Drink. Model 3: adjusted for Gender; Age; Race; BMI; Hypertension; Hyperlipemia; Diabetes.

**Fig. 3:**
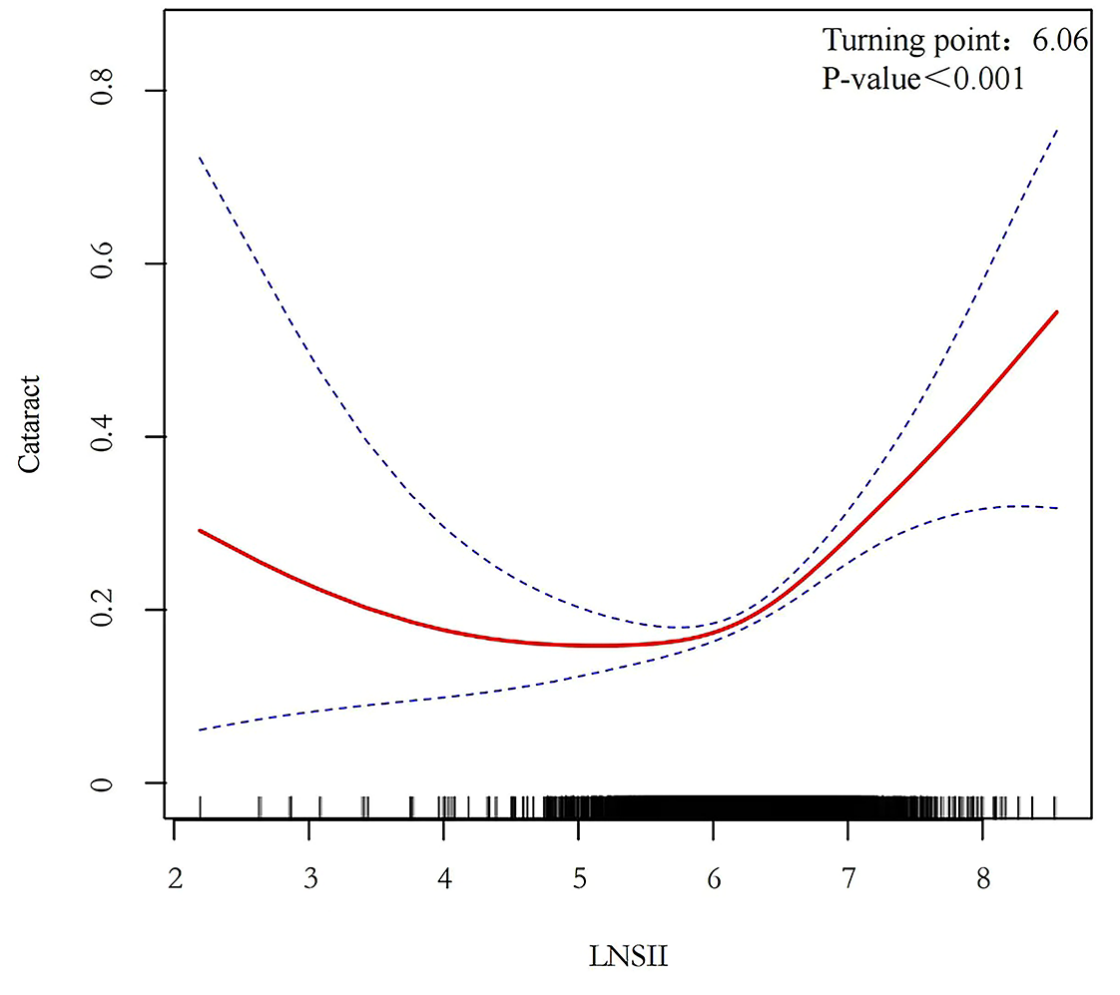
Relationship between ln-SII and cataract prevalence. Note: The red solid line represents a smoothed curve fit of SII to cataract prevalence. The blue dashed line represents the 95% confidence interval of the smoothed curve fit.

We performed a weighted multivariate logistic regression analysis (illustrated in Fig. 2), which revealed a significant positive association between elevated ln-SII scores and the likelihood of cataract prevalence. This association was consistently significant across all models: Model 1 (OR = 1.56; 95% CI = 1.33–1.85, p < 0.001), Model 2 (OR = 1.55; 95% CI = 1.32–1.84, p < 0.001), and Model 3 (OR = 1.57; 95% CI = 1.33–1.86, p < 0.001). Further analysis using smoothing spline techniques delineated a non-linear relationship between ln-SII and the odds of having cataracts across various covariates (as depicted in Fig. 3, p < 0.001). Additionally, threshold and saturation effect analyses identified an inflection point at an ln-SII of 6.06 (equivalent to an SII of 428.38). Below this threshold, the association between SII and the prevalence of cataracts is weaker; however, when SII exceeds this value, its positive correlation with the prevalence of cataracts significantly strengthens.

### Relationship of different quartiles of SII with the presence of cataract

**Fig. 4:**
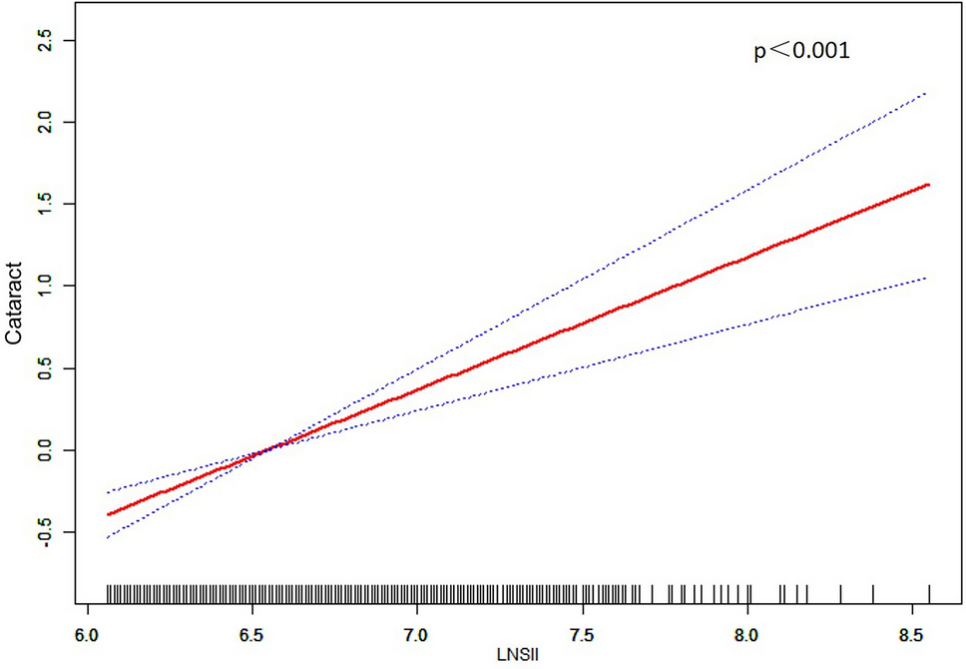
Relationship between ln-SII(values above 6.06) and cataract prevalence. *Note*: Based on the turning point (ln-SII > 6.06) in Fig. 3, the linear relationship between ln-SII and cataract adjusted for all covariates was further explored.

For sensitivity analysis, we transformed ln-SII from a continuous variable into categorical quartiles, as depicted in Table 2. Compared to the lowest quartile (Q1), participants in the highest quartile (Q4) exhibited 96% higher odds of having cataracts (OR = 1.96; 95% CI = 1.51–2.54, p < 0.001) in Model 1, 95% higher odds in Model 2 (OR = 1.95; 95% CI = 1.49–2.53, p < 0.001), and 99% higher odds in Model 3 (OR = 1.99; 95% CI = 1.53–2.59, p < 0.001). Further analysis using smoothing spline techniques demonstrated a linear relationship between ln-SII (values above 6.06) and the odds of developing cataracts, adjusted for all covariates, as illustrated in Fig. 4 (p < 0.001). This analysis substantiates the strong association between elevated ln-SII (values above 6.06) levels and increased odds of having cataracts..

**Table 2:** Association between lnSII and cataract in different quartiles.

Exposure	Model 1^a^ OR	p-Value	Model 2^b^ OR	p-Value	Model 3^c^OR	p-Value
	(95%CI)		(95%CI)		(95%Cl)	
lnSII quartiles						
Q1	1		1		1	
Q2	0.96 (0.73, 1.28)	0.792	0.96 (0.72, 1.27)	0.765	0.98 (0.74, 1.30)	0.879
Q3	1.24 (0.94, 1.63)	0.131	1.23 (0.93, 1.62)	0.145	1.25 (0.94, 1.64)	0.120
Q4	1.96 (1.51, 2.54)	< 0.001	1.95 (1.49, 2.53)	< 0.001	1.99 (1.53, 2.59)	< 0.001

^a^Model 1: adjusted for Gender; Age; Race; BMI.

^b^Model 2: adjusted for Gender; Age; Race; BMI; Education; Marital Status; Smoke; Drink.

^c^Model 3: adjusted for Gender; Age; Race; BMI; Hypertension; Hyperlipemia; Diabetes.

### PSM analysis

Given the age and numerical discrepancies between the cataract and normal groups, a 1:1 propensity score matching (PSM) analysis was conducted based on age to mitigate age-related effects. A total of 1,254 participants were enrolled and classified into the cataract and non-cataract groups. The baseline characteristics of each group, post-PSM, are presented in Table 3. Significant differences in the SII were observed between the groups after PSM, with the cataract group displaying elevated SII levels (P < 0.001). Following this, the logistic regression model, which assessed the relationship between SII and the incidence of cataracts post-PSM, indicated a significant positive correlation between higher SII scores and the prevalence of cataracts. This correlation remained consistently significant across all models (Model1:1.48 (1.21, 1.80); Model2:1.48 (1.21, 1.80); Model3:1.46 (1.20, 1.78), adhering to the inclusion criteria and details that align with those previously described, as further detailed in Supplementary Table 1.

**Table 3:** Basic characteristics of participants after PSM analysis.

Variables	Non-cataract	Cataract	*p*-Value
N	627	627	
Age (years)	73.13 ± 8.25	73.13 ± 8.25	1
BMI (kg/m^2^)	28.11 ± 5.61	28.68 ± 6.31	0.089
SII(× 10^3^/mm^n^)	602.25 ± 381.70	714.01 ± 486.36	< 0.001
Gender, n(%)			0.006
Male	333 (53.11%)	284 (45.30%)	
Female	294 (46.89%)	343 (54.70%)	
Race			0.237
Mexican	58 (9.25%)	54 (8.61%)	
Other Hispanic	34 (5.42%)	27 (4.31%)	
Non-Hispanic white	431 (68.74%)	458 (73.05%)	
Non-Hispanic black	96 (15.31%)	75 (11.96%)	
Other race	8 (1.28%)	13 (2.07%)	
Education,n(%)			0.562
Less than 9th grade	109 (17.38%)	108 (17.22%)	
9–11Th grade (includes 12th grade with no diploma)	101 (16.11%)	96 (15.31%)	
High school grad/ged or equivalent	164 (26.16%)	166 (26.48%)	
Some college or AA degre	129 (20.57%)	150 (23.92%)	
College graduate or above	124 (19.78%)	107 (17.07%)	
Marital Status,n(%)			0.279
Married or living with partner	362 (57.74%)	343 (54.70%)	
Unmarried or other	265 (42.26%)	284 (45.30%)	
Drink,n(%)			0.128
Yes	408 (65.07%)	382 (60.93%)	
No	219 (34.93%)	245 (39.07%)	
Hypertension,n(%)			0.133
Yes	365 (58.21%)	391 (62.36%)	
No	262 (41.79%)	236 (37.64%)	
Hyperlipemia,n(%)			0.125
Yes	332 (52.95%)	359 (57.26%)	
No	295 (47.05%)	268 (42.74%)	
Diabetes, n(%)			< 0.001
Yes	111 (17.70%)	170 (27.11%)	
No	516 (82.30%)	457 (72.89%)	
Smoke,n(%)			0.496
Yes	337 (53.75%)	349 (55.66%)	
No	290 (46.25%)	278 (44.34%)	

Mean ± SD for continuous variables: the p-value was calculated by a weighted linear regression model. % for categorical variables: the p-value was calculated by a weighted chi-square test. BMI, body mass index.

## Discussion

In this study, we leveraged data from the NHANES database to investigate the potential link between the SII and cataract development. Our findings from this nationally representative cross-sectional analysis revealed a nonlinear relationship between SII levels and cataract prevalence. We identified a critical inflection point for ln-SII at 6.06 (SII = 428.38), beyond which SII levels positively correlate with increased cataract prevalence. To the best of our knowledge, this is the inaugural study to explore the association between SII and cataracts, offering a novel perspective in the understanding of this relationship.

Systemic inflammation can be measured through various biochemical or hematological markers commonly assessed in routine blood analyses, or via ratios derived from these markers^22^. The SII, a novel and consistent marker of inflammation, is computed using the formula: PC × NC / LC^8^.

Elevated SII levels indicate an inflammatory environment marked by increased NC and decreased LC, potentially contributing to the onset and progression of various diseases. Such elevation not only reflects an inflammatory state but may also indicate a disruption in immune regulation. Cells like NC and LC play crucial roles in managing inflammation and immune responses. Consequently, a high SII may denote persistent immune activation, a condition frequently observed in the pathogenesis of various chronic diseases, including age-related disorders such as cataracts^23^.

The connection between inflammatory states and cataract formation may be mediated by inflammatory cytokines, such as tumor necrosis factor-alpha (TNF-α) and interleukin-6 (IL-6)^24^. Released during inflammatory responses, these cytokines directly alter the intraocular environment, promoting oxidative damage and apoptosis in lens epithelial cells, thereby accelerating the development of cataracts. Concurrently, elevated levels of SII lead to an increase in inflammatory mediators within the bloodstream. These mediators, penetrating the eye via ocular circulation, trigger inflammatory pathways within the lens. This activation induces further cellular responses, including the release of additional cytokines, which exacerbate oxidative stress and cellular damage in the lens^25^.

Alterations in the SII may also indicate the infiltration status of immune cells, particularly NC and LC, within the eye. The activation and subsequent infiltration of these cells can directly damage the lens or indirectly lead to lens cell injury and death through the release of inflammatory mediators and enzymes. Such processes significantly accelerate the development of cataracts.

Oxidative stress is intricately linked to mitochondrial function. Under conditions of inflammation and elevated SII, oxidative stress can escalate, leading to mitochondrial dysfunction—a key factor in the development of cataracts. This dysfunction may decrease ATP production and disrupt cellular metabolism, impairing the normal functioning and viability of lens cells. Furthermore, mitochondrial dysfunction can enhance the production of reactive oxygen species (ROS) within cells, further exacerbating oxidative stress^26^.

Additionally, prolonged inflammatory responses and oxidative stress can deplete the body’s antioxidant defense mechanisms. Notably, the activities of antioxidant enzymes, such as superoxide dismutase (SOD) and glutathione peroxidase (GP), may diminish. Concurrently, the overall levels of antioxidants may decrease, impairing the eye’s capacity to neutralize ROS. This reduction in antioxidant defense can lead to increased oxidative damage to the lens, exacerbating conditions conducive to cataract formation.

Considering the established correlation between the SII and cataracts, future research should focus on therapeutic strategies that target inflammation and oxidative stress. Potential approaches could include the development of novel pharmacological agents or nutritional supplements designed to mitigate inflammatory responses, enhance antioxidant defenses, or directly scavenge ROS. Additionally, subsequent studies ought to evaluate the utility of SII in clinically assessing cataract risk and monitoring disease progression. With an enhanced understanding of the precise relationship between SII and cataract formation, SII could serve as a pivotal marker for predicting cataract risk and directing appropriate intervention strategies.

Our study is bolstered by a substantial, nationally representative sample and adjusts for critical demographic, examination, and laboratory factors, thereby enhancing the credibility and generalizability of our findings. However, the study also exhibits limitations, chiefly its cross-sectional design, which constrains our capacity to establish causality. Furthermore, the NHANES survey administrators did not thoroughly account for the age-specific characteristics of cataracts, including comprehensive records of bilateral conditions during ophthalmological screenings. To address these challenges, future research employing a robust sample size is essential to clarify causal relationships. Additionally, it is imperative to further investigate the possible association between SII and the history of bilateral cataract surgery.

## Conclusion

This study, drawing on data from the NHANES database, unveils new insights into the correlation between the SII and cataract development. Typically, SII is positively correlated with inflammatory conditions, such as cataract. A critical SII value of 428.38 emerges with significant clinical implications. These findings propose SII as a novel biomarker for the risk assessment and early prevention of cataracts, highlighting the pivotal role of systemic inflammation in cataract pathogenesis.

## Supplementary Information

Supplementary Information.

## Data Availability

Publicly available datasets were analyzed in this study. This data can be found at: https://www.cdc.gov/nchs/nhanes/index.htm.

## Abbreviations

SII: Systemic immune-inflammation index
NHANES: National health and nutrition examination survey
PSM: Propensity score matching
CBC: Complete blood count
WBC: White blood cell
MEC: Mobile examination center
BMI: Body mass index
SE: Standard errors
OR: Odds ratio
TNF-α: Tumor necrosis factor-alpha
IL-6: Interleukin-6
ROS: Reactive oxygen species
SOD: Super oxide dismutase
GP: Glutathione peroxidase
LC: Lymphocytes
NC: Neutrophils
PC: Platelets
PCME: Pseudophakic cystoid macular edema
ln: Logarithmic
