# Supplementary material for: Association between systemic immune inflammation index and cataract incidence from 2005 to 2008
Source: Sci Rep. 2025 Jan 2;15:499. doi: 10.1038/s41598-024-84204-7 (PMC11696561; doi:10.1038/s41598-024-84204-7)
Supplement: Supplementary file 1 — Supplementary Information. [file 41598_2024_84204_MOESM1_ESM.docx]

| Supplement Table 1. Association between lnSII(values above 6.06) and cataract. | | | | | | |
| --- | --- | --- | --- | --- | --- | --- |
| **Exposure** | **Model** **1^a^** **OR** | **p-Value** | **Model 2^b^** **OR** | **p-Value** | **Model** **3^c^OR** | **p-Value** |
|  | （**95%CI）** |  | **(95%CI）** |  | （**95%Cl)** |  |
| lnSII | 1.48 (1.21, 1.80) | 0.0001 | 1.48 (1.21, 1.80) | 0.0001 | 1.46 (1.20, 1.78) | 0.0002 |
| ^a^Model 1: adjusted for Gender; Age; Race; BMI | | | | | | |
| ^b^Model 2: adjusted for Gender; Age; Race; BMI; Education; Marital Status; Smoke; Drink | | | | | | |
| ^c^Model 3: adjusted for Gender; Age; Race; BMI; Hypertension; Hyperlipemia; Diabetes | | | | | | |
